# Medical Comics as Tools to Aid in Obtaining Informed Consent for Stroke Care

**DOI:** 10.1097/MD.0000000000001077

**Published:** 2015-07-02

**Authors:** Yuichi Furuno, Hiroyasu Sasajima

**Affiliations:** From Department of Neurosurgery, Graduate School of Medical Science, Kyoto Prefectural University of Medicine, Kyoto, Japan (YF, HS)

## Abstract

Informed consent has now become common in medical practice. However, a gap still exists between doctors and patients in the understanding of clinical conditions. We designed medical comics about “subarachnoid hemorrhage” and “intracerebral hemorrhage” to help doctors obtain informed consent intuitively, quickly, and comprehensively.

Between September 2010 and September 2012, we carried out a questionnaire survey about medical comics with the families of patients who had suffered an intracerebral or subarachnoid hemorrhage. The questionnaire consisted of 6 questions inquiring about their mental condition, reading time, usefulness of the comics in understanding brain function and anatomy, pathogenesis, doctor's explanation, and applicability of these comics.

The results showed that 93.8% responders would prefer or strongly prefer the use of comics in other medical situations. When considering the level of understanding of brain function and anatomy, pathology of disease, and doctor's explanation, 81.3%, 75.0%, and 68.8% of responders, respectively, rated these comics as very useful or useful.

We think that the visual and narrative illustrations in medical comics would be more helpful for patients than a lengthy explanation by a doctor. Most of the responders hoped that medical comics would be applied to other medical cases. Thus, medical comics could work as a new communication tool between doctors and patients.

## INTRODUCTION

Informed consent has now become common in medical practice. However, a gap still exists between doctors and patients in understanding clinical conditions. Emergency conditions such as strokes widen this gap, as patients and their family have difficulty understanding technical terms under time constraints. We created medical comics about “subarachnoid hemorrhage” and “intracerebral hemorrhage,” which illustrated the pathogenesis, clinical condition, treatment, and prognosis including rehabilitation of the disease. These comics may help doctors obtain informed consent intuitively, quickly, and comprehensively. However, empirical research examining the advantages and disadvantages of these medical comics has never been conducted.

The purpose of this study was to determine whether medical comics facilitate in obtaining informed consent during an emergency condition. In order to evaluate our methodology, we carried out a questionnaire survey to investigate how these comics could help the patient in terms of time efficiency, level of understanding, and applicability to other medical cases.

## METHODS

Between September 2010 and September 2012, we carried out a questionnaire survey with the families of patients admitted to the Department of Neurosurgery, University Hospital, Kyoto Prefectural University of Medicine for intracerebral or subarachnoid hemorrhage. After initial management of the patients in the emergency room, we explained the medical condition, necessary examinations, treatment, and predictable prognosis to the patient's family with the help of pictures in medical comics. We then gave them medical comics, which were 25 pages long for “subarachnoid hemorrhage” and 35 pages long for “intracerebral hemorrhage,” and requested them to read them and answer the questionnaire survey. The questionnaire consisted of 6 questions inquiring about their mental condition, reading time, usefulness of medical comics in understanding brain function and anatomy, pathogenesis, doctor's explanation, and applicability of medical comics (Table 1). We utilized a five-level rating system, except for reading time, where we utilized a four-level rating system.

**TABLE 1:**
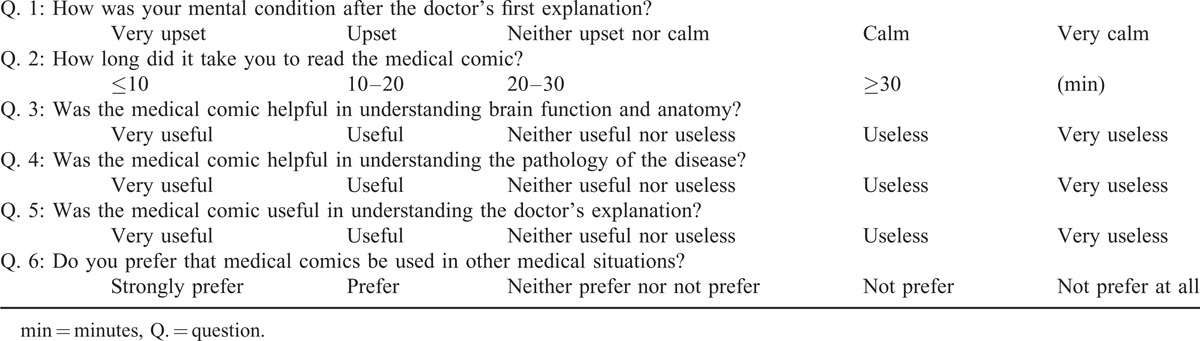
Questionnaire Survey About Medical Comics

This study was approved by the ethics committee of Kyoto Prefectural University of Medicine. We gave detailed descriptions of the study to each patient's family, and all participants signed informed consent forms before participation.

## RESULTS

The survey was answered by a total of 16 people, 7 males and 9 females (1 or 2 individuals from each patient's family), with an average age of 46.9 years (range: 26–68 years; Table 2). The results showed that 93.8% of the responders read medical comics in <30 min, and would prefer or strongly prefer that they be applied to other medical cases. When considering the level of understanding of brain function and anatomy, pathology of disease, and doctor's explanation, 81.3%, 75.0%, and 68.8%, respectively, rated medical comics as very useful or useful (Table 3).

**TABLE 2:**
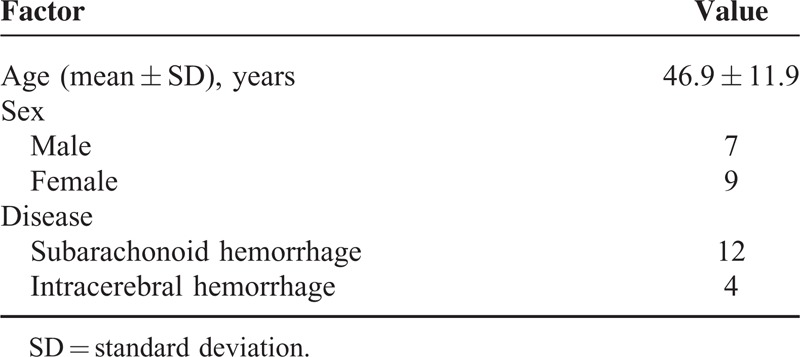
Summary of 16 Responders

**TABLE 3:**
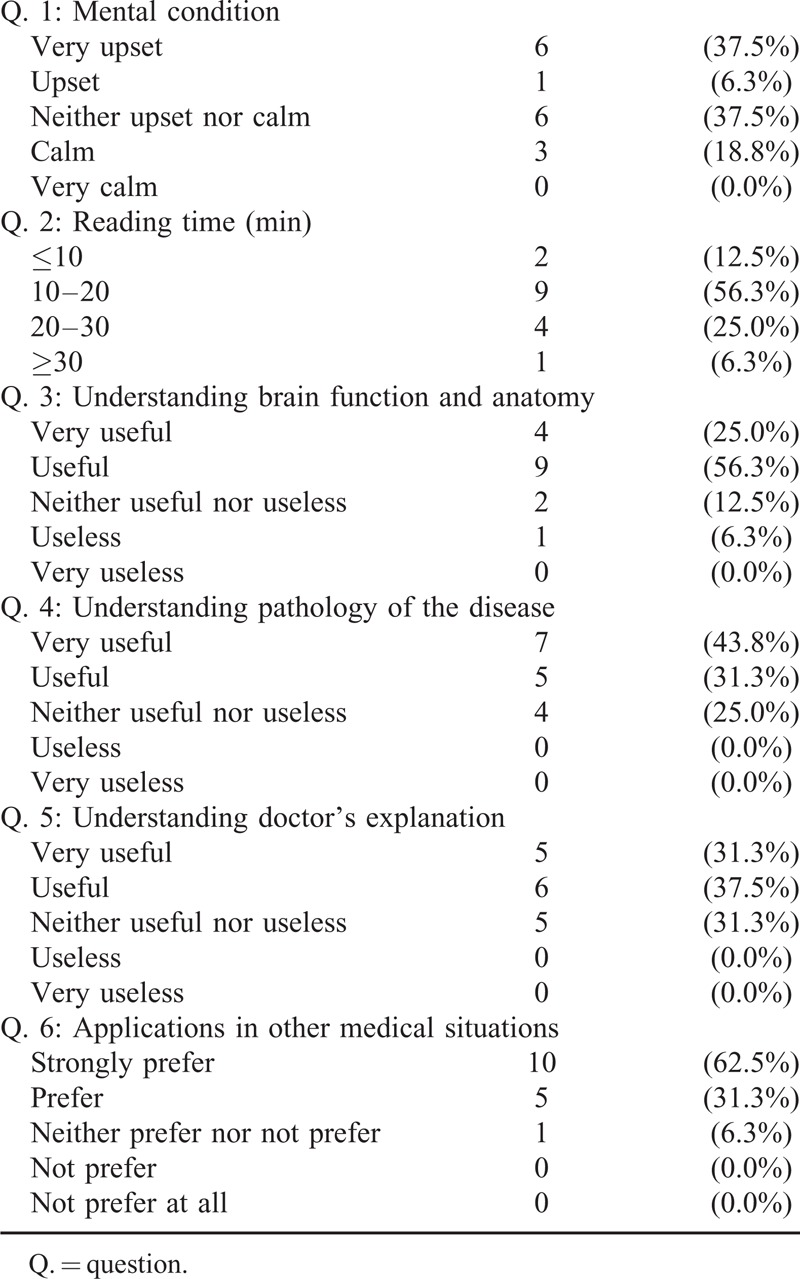
Results of the Questionnaire Survey (Q. 1–6)

## DISCUSSION

According to our survey, medical comics seemed to have the capability to facilitate in obtaining informed consent during emergency conditions, such as strokes, as they encourage the patients and their families to understand the facts, implications, and future consequences of an action within a short period of time. We were able to obtain good results for the level of understanding of brain function and anatomy, pathology of disease, and doctor's explanation. However, the responses received were unsatisfactory, particularly for doctor's explanation. Almost all the readers hoped that medical comics like these would be utilized in other medical cases. Therefore, we must keep improving them and continue our research in this field.

Comics have evolved over the past 100 years^1^ and have become a popular source of amusement for both children and adults, particularly in Japan. They have also become a prominent part of the media in recent years. Government offices and self-governing bodies have also adopted comics as public relation tools.^2,3^ Moreover, they can serve educational purposes by combining pictures and words and giving visual cues to what the text is explaining.^4–8^ They also have a remarkable capacity to convey information visually, attract the interest of readers, and are overall pleasurable to read.^4^ Moreover, the contents tend to remain in the memory for a longer period of time because of their contents in the form of a story.

**FIGURE 1:**
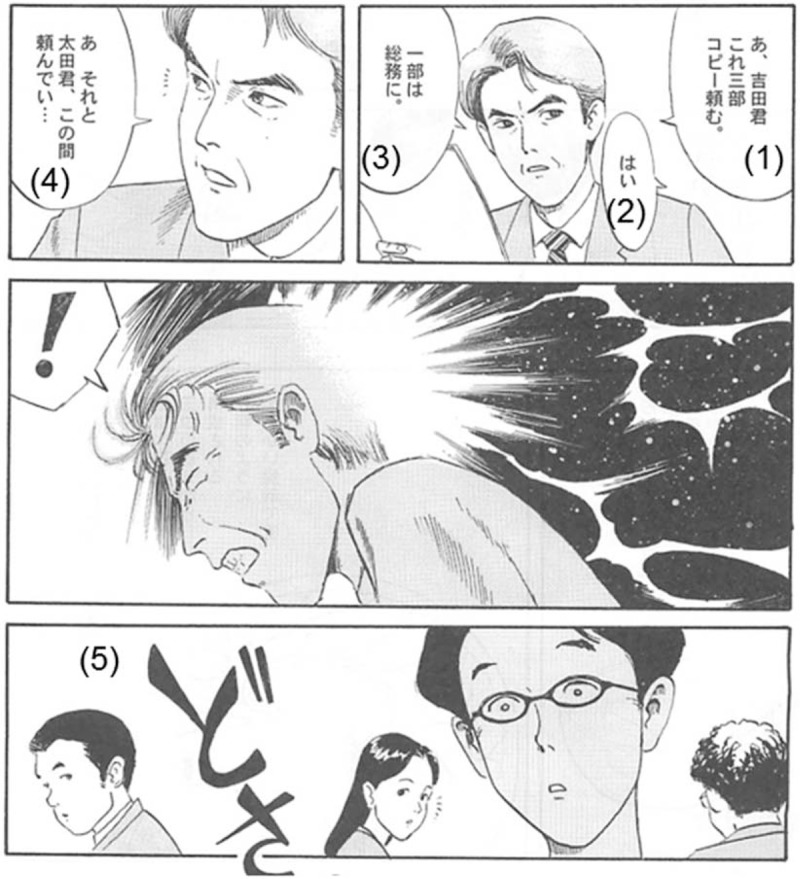
This page depicts the scene in which a middle-aged man in the prime of his working life begins to experience sudden severe headaches. Translation: (1) Mr. Yoshida, I’d like two copies of this document. (2) Yes. (3) Take one of those to the Department of General Affairs. (4) Well, Mr. Ota, let me know how … (5) Bang!.

**FIGURE 2:**
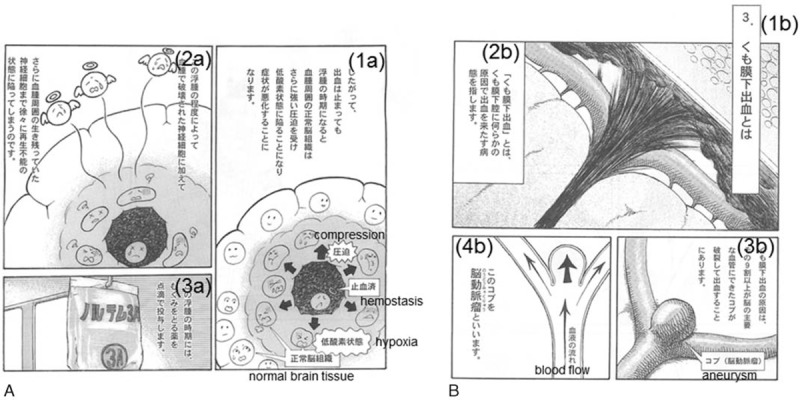
These pages explain the pathogenesis. (A) Explains how intracerebral hematoma destroys perihematomal neurons and (B) the image of how aneurysms rupture. Translation: (1a) Therefore, although it stops bleeding, symptoms will worsen during periods of perihematomal edema, which compress normal tissue strongly and make it hypoxic. (2a) Perihematomal edema kills more normal perihematomal tissue. (3a) We administer patients an intravenous drip injection, which improves brain edema during periods of perihematomal edema. (1b) What is subarachnoid hemorrhage? (2b) A subarachnoid hemorrhage is defined as bleeding into the subarachnoid space for some reason. (3b) This may occur usually from a ruptured blood-filled balloon-like bulge in the wall of a blood vessel. (4b) We call it an aneurysm.

**FIGURE 3:**
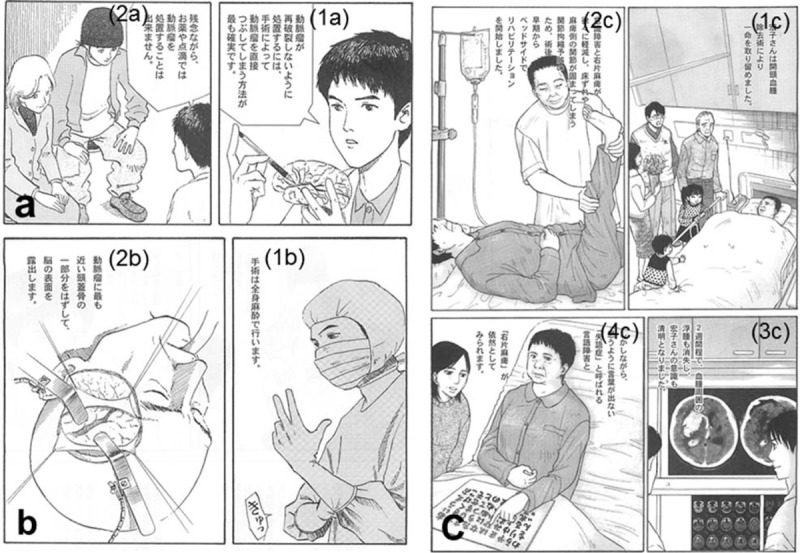
Medical comics have real stories about the diseases’ clinical course. Scenes of doctors explaining (A) surgical treatment and (B) rehabilitations (C). Translation: (1a) Surgical clipping of the aneurysm is the most certain means of preventing rebleeding. (2a) Unfortunately, drugs are not effective to manage the aneurysm. (1b) The surgery is performed under general anesthesia. (2b) Depending on the location of the aneurysm, craniotomy is performed and the brain tissue is exposed. (1c) Ms. Hiroko escaped death thanks to the craniotomy for hematoma removal. (2c) She gained complete consciousness and gradual improvement of right hemiparesis. She started rehabilitation early to prevent bedsores and joint contractures. (3c) Perihematomal edema disappeared approximately 2 weeks after onset. Ms. Hiroko was completely consciousness. (4c) However, she still had right hemiparesis and aphasia which made it difficult for her to say what she meant to say.

In medical practice, comics are used to provide explanations, particularly to children with low levels of literacy and immigrants.^4,9–14^ Medical comics can be helpful to patients wanting to learn more about their illness. Their powerful visual messages convey immediate pictorial understanding in ways that conventional texts cannot. However, this is the first time these medical comics have been tried in an emergency situation, and the patient's families can use them to educate themselves on the continuum of care. The peak age of onset and background of patients are reflected in the characters of our medical comics. For example, the “subarachnoid hemorrhage” comic begins with a middle-aged man, in the prime of his working life, suffering from sudden severe headaches (Figure 1). The readers can know the situation easily from the pictures and their lines. Further descriptions are provided in auxiliary text, so that readers can deepen their understanding. Deformed pictures are inserted in explanations for anatomy or pathogenesis, so that readers can be sociable to them (Figure 2). We think that these familiarities would be effective in high stress situations, such as in an emergency room. These books have stories that include onset, doctor's explanations, treatments, and rehabilitation for the disease (Figure 3). The explanation of rehabilitation is very important as most patients’ families are often worried about the patient's functional prognosis at the time of first visit. Almost all participants of this survey were able to read the medical comics in less than 30 min. Another advantage of medical comics is that the readers can read them repeatedly, after they hear the explanation from the doctors.

We think the visual and narrative illustration in the medical comics would be more helpful for patients and their families, than a lengthy explanation conducted by a doctor. Medical comics could work as a new communication tool for informed consent between doctor and patient.
